# Rupture of the Abdominal Aorta without Aneurysm Associated with Giant Cell Arteritis: A Case Report

**DOI:** 10.3400/avd.cr.25-00010

**Published:** 2025-06-03

**Authors:** Akito Kuwano, Masaru Yoshikai, Satoshi Ohtsubo, Kiyokazu Koga, Nozomi Yoshida, Naoyo Nishida

**Affiliations:** 1 Department of Cardiovascular Surgery, Shin-Koga Hospital, Kurume, Fukuoka, Japan; 2 Department of Pathology, Shin-Koga Hospital, Kurume, Fukuoka, Japan

**Keywords:** giant cell arteritis, abdominal aorta, rupture

## Abstract

We report a case of an abdominal aortic rupture without aneurysm associated with giant cell arteritis. A 67-year-old woman presented with lower back pain. Contrast-enhanced computed tomography revealed a massive retroperitoneal hematoma with contrast leakage from the abdominal aorta, suggestive of abdominal aortic rupture. During emergency surgery, a rupture site was identified on the anterior wall of the abdominal aorta, while no aneurysmal changes or dilatation of the abdominal aorta were observed. Histopathological examination of the resected aortic wall revealed infiltration of giant cells positive for CD68, leading to the diagnosis of giant cell arteritis.

## Introduction

Giant cell arteritis (GCA) is a systemic inflammatory disease that predominantly affects elderly women. While the superficial temporal artery is the most commonly affected site, GCA can also lead to aortic dissection or aneurysm.^1,2)^ The prevalence of aortic dissection and aortic aneurysm has been reported to be 17 times higher in the thoracic aorta and 2.4 times higher in the abdominal aorta compared to healthy individuals.^2)^ However, cases of abdominal aortic rupture without aneurysm associated with GCA have never been reported. We performed an emergency open surgery for such a case of abdominal aortic rupture, leading to the diagnosis of GCA.

## Case Report

**Figure 1:**
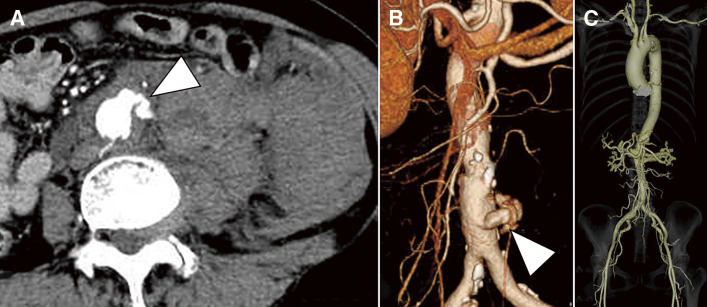
Fig. 1 Preoperative CT findings. Preoperative contrast-enhanced CT reveals extravasation of the contrast from the infrarenal abdominal aorta (arrowhead), accompanied by a massive retroperitoneal hematoma (**A**). No apparent aneurysmal changes, dilatation, or stenosis were observed in the entire aorta, including the thoracic aorta, abdominal aorta, and their branches (**B**, **C**). CT: computed tomography

A 67-year-old woman with no significant medical history presented to our facility with the sudden onset of lower back pain. Upon presentation, the patient was in a state of shock, with a blood pressure of 65/38 mmHg and a pulse rate of 86 bpm in sinus rhythm. Her abdomen was distended and exhibited tenderness. Although she was afebrile, blood laboratory tests revealed an elevated white blood cell count of 26900/μL, while C-reactive protein (CRP) was negative. Serum immunoglobulin G4 (IgG4) levels and other antibody tests for immune disorders were within the normal range; however, erythrocyte sedimentation rate (ESR) was not measured. Contrast-enhanced computed tomography (CT) revealed extravasation of the contrast from the infrarenal abdominal aorta, accompanied by a massive retroperitoneal hematoma. However, no apparent aneurysmal changes, dilatation, or stenosis were observed in the entire aorta, including the thoracic aorta, abdominal aorta, and their branches (**Fig. 1**). The diagnosis of the abdominal aortic rupture prompted us to perform an emergency open surgery.

**Figure 2:**
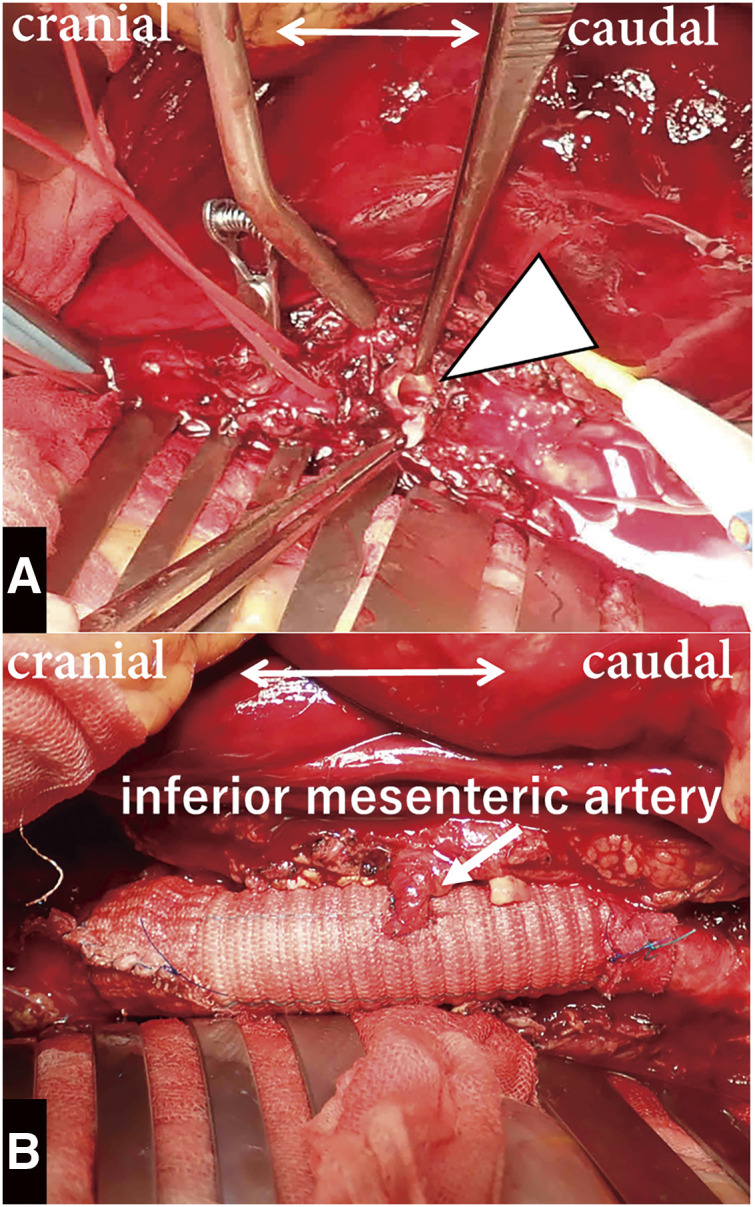
Fig. 2 Intraoperative findings. Intraoperative findings reveal a rupture site on the anterior wall of the abdominal aorta, with a defect approximately 8 mm in size (arrowhead) (**A**), along with photographs taken after the completion of the surgical procedure (**B**).

**Figure 3:**
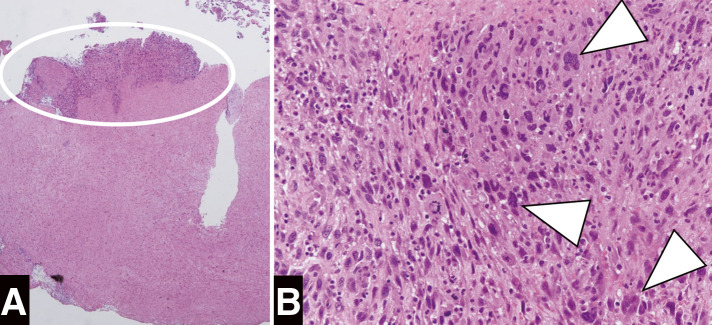
Fig. 3 Histopathological examination of the resected aortic wall. Histopathological examination reveals infiltration of giant cells positive for CD68 (arrowhead), without atherosclerotic changes. (**A**) Magnification: 20x, hematoxylin and eosin staining. (**B**) Magnification: 200x, hematoxylin and eosin staining.

During the surgery, after a midline abdominal incision, bloody ascites was observed, and the retroperitoneum was filled with a massive hematoma (Fitzgerald classification IV). An approximately 8-mm-sized defect was detected on the anterior wall of the abdominal aorta, which was identified as a rupture site (**Fig. 2A**). Aneurysmal dilatation of the abdominal aorta was not observed. The abdominal aorta, from its infrarenal portion to the abdominal bifurcation, was replaced with a straight graft (J-graft 14 mm; Japan Lifeline, Tokyo, Japan), and the inferior mesenteric artery was reconstructed. The proximal anastomosis was wrapped with a graft material (**Fig. 2B**). The patient recovered uneventfully and was discharged on postoperative day 9. Histopathological examination of the resected aortic wall revealed that inflammation primarily involved the intima and media, with infiltration of CD4-positive T cells and CD68-positive giant cells. No signs of atherosclerotic changes were observed, supporting the diagnosis of GCA (**Fig. 3**). The postoperative carotid artery ultrasound examination revealed no significant findings. Contrast-enhanced CT showed no apparent aneurysmal changes, dilatation, or stenosis in the entire aorta and its branches; therefore, magnetic resonance imaging was not performed. As CRP levels remained negative at the time of the definitive diagnosis of GCA, indicating an inactive phase of inflammation, positron emission tomography-CT (PET-CT) was not performed, and corticosteroid therapy was not initiated. One year after the surgery, CRP levels have remained negative and the patient is doing well without any aortic-related events.

## Discussion

GCA is a systemic inflammatory disease that typically affects women over the age of 50. Although the superficial temporal artery is the most commonly affected site, GCA can also cause stenosis of the carotid and subclavian arteries, aortic aneurysms, and aortic dissection.^1,2)^ According to the 2022 American College of Rheumatology classification criteria, morning stiffness in the shoulder or neck, sudden visual loss, jaw or tongue claudication, new temporal headache, scalp tenderness, and abnormal examination of the temporal artery are highlighted as representative clinical symptoms of GCA.^3)^ In this case, the patient had no specific symptoms related to GCA preoperatively, and histopathological examination of the resected aortic wall revealed infiltration of giant cells. Differential diagnoses for vasculitis include Takayasu arteritis and IgG4-related disease. The hallmark histopathological findings of Takayasu arteritis are panarteritis with marked fibrosis, while IgG4-related disease is characterized by a prominent infiltration of IgG4-positive plasma cells. In this case, these characteristic histopathological features were absent, and the giant cell infiltration was predominantly confined to the media, ultimately leading to the diagnosis of GCA. In cases of asymptomatic GCA with aortic involvement, the diagnosis of GCA can be made solely based on the histopathological examination of the vessel wall.^4)^

There have been only a few reports of abdominal aortic rupture associated with GCA. Kwon et al. reported a case of contained rupture of an abdominal aortic aneurysm.^5)^ Another case of an abdominal aortic aneurysm rupture associated with GCA was diagnosed on an autopsy.^6)^ Both of these cases exhibited aneurysmal changes in the abdominal aorta; however, the present case did not demonstrate any aneurysmal changes or dilatation of the abdominal aorta. Therefore, this evidence highlights the possibility of aortic rupture due to GCA, even in the absence of aneurysmal changes or aortic dilatation. The potential causes of vascular fragility in vasculitis include the degradation of elastin and collagen through the activation of matrix metalloproteinases, medial necrosis, endothelial damage, and immunological mechanisms mediated by the activation of CD4-positive T cells. In this case, histopathological findings revealed extensive inflammation, yet the characteristic features of elastin fiber rupture, medial necrosis, and endothelial injury were not clearly evident. Given the marked infiltration of CD4-positive T cells within the inflamed regions, it is hypothesized that vascular fragility was induced through immunological mechanisms, thereby contributing to the rupture of the aorta without aneurysm.

The differential diagnosis of non-atherosclerotic aortic diseases without aneurysmal changes or dilatation includes inflammation, infection, trauma, and tumors. In this case, there was no history of trauma, nor were there preceding episodes of infection or fever, making the involvement of trauma or infection unlikely. On the other hand, differentiating inflammatory aortic diseases, including GCA, Takayasu arteritis, and IgG4-related disease, from aortic tumors based solely on contrast-enhanced CT findings remains challenging. In patients without aortic aneurysm or dilatation but exhibiting characteristic symptoms of GCA, a temporal artery biopsy is essential for confirming the diagnosis. Measurement of CRP and ESR, along with systemic imaging such as PET-CT, should be performed to assess vasculitis activity. In patients in the active phase, corticosteroid and immunosuppressive therapy are considered essential to prevent future aortic involvement associated with GCA. However, in the present case, which was asymptomatic and required emergency surgery, there was insufficient time for a comprehensive preoperative evaluation. Considering the patient's age (over 50 years) and a serum IgG4 level within the normal range, the probability of GCA was deemed to be high, and the diagnosis was ultimately confirmed through histopathological examination. Given the difficulty of making a differential diagnosis based solely on clinical and imaging findings, in cases where non-atherosclerotic aortic diseases without aneurysmal changes or dilatation are suspected and preoperative differentiation of the etiology is challenging, direct visualization of the aorta during surgery, along with histopathological examination of the aortic wall, is essential for establishing a definitive diagnosis and formulating an appropriate postoperative management plan. When endovascular aortic repair (EVAR) is chosen instead of open surgery, a definitive diagnosis of GCA would not be possible, which could potentially impact the postoperative treatment strategy and prognosis.

There have been reports of complications following thoracic aortic graft replacement in patients with GCA, including anastomotic disruption and the formation of a fistula between the right atrium and a synthetic graft.^4,7)^ It is important to be cautious of aortic-related events following surgical intervention; however, there is no definitive evidence regarding postoperative management, including corticosteroid administration, for aortic involvement associated with GCA. Reports on GCA and postoperative aortic-related events are limited, and it has been suggested that the management approach for GCA patients should follow that of Takayasu arteritis.^8)^ According to the 2021 American College of Rheumatology/Vasculitis Foundation guideline, the use of high-dose glucocorticoids is conditionally recommended during the periprocedural period if the patient is in the active phase.^8)^ Meanwhile, Fields et al. reported that in patients who underwent surgical treatment for Takayasu arteritis, freedom from graft revision at 5 and 10 years was 100% in those with quiescent disease who did not require steroid therapy.^9)^ On the other hand, corticosteroid therapy following cardiovascular surgery could cause systemic infections, including wound and graft infections. Therefore, it is essential to carefully assess the activity of vasculitis and comorbidities in each individual to determine whether postoperative corticosteroid therapy should be initiated. In this case, the diagnosis of GCA had not been established preoperatively, and as CRP levels remained negative at the time of the definitive diagnosis of GCA, suggesting quiescent disease, corticosteroid therapy was not initiated. Following this approach, for 1 year after surgery, CRP levels have remained negative, and no aortic-related events have occurred. However, continued careful follow-up will be necessary moving forward. Should inflammation become activated, such as an elevation in CRP levels, we intend to initiate corticosteroid therapy. Routine imaging follow-up is recommended for Takayasu arteritis, even in clinically quiescent cases; however, there is no definitive evidence regarding the optimal imaging follow-up interval for GCA.^8)^ In cases with active inflammation at the time of diagnosis, a shorter follow-up interval is warranted, whereas a longer interval may be appropriate once the disease is quiescent. Given the risk of aortic-related complications, such as anastomotic disruption, and the necessity of careful long-term monitoring, EVAR may be considered a first-line treatment option for an abdominal aortic aneurysm with a preoperative diagnosis of GCA.

In this case, CRP levels remained negative, indicating an inactive phase of inflammation; therefore, PET-CT was not performed. However, Peyrac et al. reported an association between ^18^F-fluorodeoxyglucose uptake in limb arteries on PET-CT and GCA relapse.^10)^ Therefore, PET-CT should have been used after the diagnosis of GCA to assess systemic inflammation. When significant uptake is observed, corticosteroid therapy is recommended to prevent relapse and aortic-related complications, including postoperative anastomotic disruption.

## Conclusions

We present a case of abdominal aortic rupture associated with GCA. This case demonstrates that aortic rupture can occur in GCA without aneurysm formation. In cases of asymptomatic GCA with aortic involvement, where the diagnosis has not been established, surgical intervention may be considered a first-line treatment option, and histopathological examination of the resected aortic wall remains the only method to establish a definitive diagnosis.

## Declarations

### Informed consent

Informed consent was obtained from this patient.

### Acknowledgments

None.

### Disclosure statement

The authors declare that there is no conflict of interest.

### Author contributions

Study conception: AK and MY

Data collection: AK and MY

Analysis: AK and MY

Investigation: AK and MY

Manuscript preparation: AK and MY

Funding acquisition: None

Critical review and revision: all authors

Final approval of the article: all authors

Accountability for all aspects of the work: all authors.
